# Endpoints of Periodontal Therapy in Elderly Patients With Stage III/IV Periodontitis and Their Oral Health–Related Quality of Life Following 10 Years of Supportive Periodontal Therapy

**DOI:** 10.1111/jcpe.14198

**Published:** 2025-07-06

**Authors:** Caspar Victor Bumm, Sophie Gaenesch, Florian Nagler, Iris Frasheri, Falk Schwendicke, Vinay Pitchika, Christina Ern, Richard Heym, Charlotte Wetzel, Matthias Folwaczny, Nils Werner

**Affiliations:** ^1^ Department of Conservative Dentistry, Periodontology and Digital Dentistry University Hospital, LMU Munich Munich Germany; ^2^ Private Practice Munich Germany

**Keywords:** multivariable analysis, oral health–related quality of life, periodontitis

## Abstract

**Aim:**

To investigate clinical endpoints of periodontal therapy after steps 1 and 2 of therapy and their association with oral health–related quality of life (OHRQoL) following long‐term supportive periodontal therapy (SPT).

**Materials and Methods:**

Forty‐seven patients receiving SPT for 126 ± 30 months were included. Clinical endpoints of therapy, as proposed by the EFP (PPD ≤ 3 mm, ≤ 5 mm without bleeding on probing), and a treat‐to‐target endpoint (T2T; ≤ 4 sites with PPD of ≥ 5 mm) were determined following steps 1 and 2 of therapy (T1) and were associated with patients' OHRQoL using the Oral Health Impact Profile (OHIP)‐14 as well as tooth loss (TL) and self‐reported tooth migration 126 ± 30 months after step 2 (T2).

**Results:**

One patient achieved the EFP endpoint and 16 achieved T2T, and 30 patients failed to achieve any endpoint at T1. OHRQoL at T2 did not differ significantly between patients achieving or not achieving the endpoints (*p* = 0.485). Self‐reported tooth migration during the examination period was significantly associated with poorer OHRQoL (*p* = 0.009).

**Conclusions:**

OHRQoL has become a major subject of periodontal research. Within the limitations of this study, achieving clinical endpoints does not seem to affect patients' OHRQoL following long‐term SPT. Patients reporting on tooth migration, however, showed significantly reduced OHRQoL. Besides clinical endpoints, functional and aesthetic complaints of periodontally compromised patients should be considered when evaluating the success of therapy.

## 1 Introduction

Oral health–related quality of life (OHRQoL) is an essential part of oral health reflecting its impact on daily life and overall well‐being. It encompasses physical, psychological and social aspects of living that are influenced by oral health, including the ability to eat, speak and interact with others without discomfort or embarrassment.

Historically, the assessment of oral health has evolved from clinical measures to patient‐centred evaluations (Sischo and Broder 2011). While early tools (Cushing et al. 1986; Lang et al. 1997) recognised oral health's broader effects, they lack comprehensive patient perspectives. In this context, the Oral Health Impact Profile (OHIP) (Slade and Spencer 1994) marked a major advancement, systematically measuring functional, psychological and social impacts, with its shorter version, the OHIP‐14 (Slade 1997), remaining one of the most widely used OHRQoL tools today (John et al. 2016).

Regardless of the methodology of its assessment, OHRQoL is a multifaceted construct providing a comprehensive measure of the outcomes of oral health interventions beyond the traditional clinical indicators, offering a more patient‐centred approach to evaluate oral health conditions (Sischo and Broder 2011; Graziani and Tsakos 2020).

Among these conditions, periodontitis is a highly prevalent non‐communicable disease affecting the supporting structures of the teeth and represents an increasing global health burden (Kassebaum et al. 2014; Petersen and Ogawa 2012; Nascimento et al. 2024). In patients affected by periodontitis, the dysbiotic subgingival biofilm is known to cause dysregulated and destructive host immune responses that are influenced by various genetic, epigenetic and environmental factors (Hajishengallis et al. 2020; Larsson et al. 2015; Reynolds 2014; Bagaitkar et al. 2011; Offenbacher et al. 2016; Xiao et al. 2017; Hajishengallis 2014; Joshi et al. 2014).

Considered the major causes of tooth loss (TL) in adults in high‐income countries, periodontitis affects not only nutrition but also the quality of life (QoL) and self‐esteem (Tonetti et al. 2017). Various symptoms of periodontitis, such as increased tooth mobility, occlusal trauma, flaring/drifting, TL and associated diseases such as halitosis may induce pain, discomfort or aesthetic concerns. These symptoms can adversely affect daily activities such as eating and speaking, but may also have psychological and social consequences, including decreased self‐esteem, social withdrawal and reduced life satisfaction (Buset et al. 2016; Shanbhag et al. 2012; Ng and Leung 2006). Furthermore, the partially bidirectional association between periodontitis and various systemic conditions, such as cardiovascular diseases, atherosclerosis, diabetes mellitus, rheumatoid arthritis, pulmonary infections and adverse pregnancy outcomes (Tonetti et al. 2017; Sanz, Marco del Castillo, et al. 2020; Schenkein et al. 2020; Sanz and Kornman 2013; Preshaw et al. 2012; Kruse et al. 2018), may exacerbate the impact of periodontitis on OHRQoL.

Despite the demand for tangible patient‐reported outcome measures and growing evidence that suggests a clear association between periodontitis and OHRQoL (Graziani and Tsakos 2020; Loos and Needleman 2020), several recently published endpoints of periodontal therapy, indicating periodontal stability or further need for active periodontal treatment (APT), heavily rely on clinical outcome measures (Chapple et al. 2018; Sanz, Herrera, et al. 2020; Feres et al. 2020). While the European Federation of Periodontology (EFP) published expert, consensus‐based recommendations for the treatment of stages I‐III periodontitis, setting rather strict thresholds to achieve the clinical endpoints of APT (Sanz, Herrera, et al. 2020), the treat‐to‐target (T2T) endpoint introduced by Feres et al. allows for residual periodontal pocketing up to a certain number of teeth (Feres et al. 2020).

Besides long‐term data on the effect of supportive periodontal therapy (SPT) on OHRQoL (Vogt et al. 2023; Sonnenschein et al. 2018; Graetz et al. 2020; El Sayed et al. 2019), little evidence is available on the validity of clinical endpoints to also reflect the OHRQoL of an individual. Therefore, the aim of this study was to investigate different endpoints of periodontal therapy (Sanz, Herrera, et al. 2020; Feres et al. 2020) and their long‐term impact on disease stability and OHRQoL, testing the null hypothesis that clinical outcomes of periodontal therapy are not associated with OHRQoL following long‐term SPT.

## 2 Methods

This longitudinal follow‐up study was approved by the Ethics Committee of the Medical Faculty of the Ludwig‐Maximilians‐University, Munich (no. 025‐11), and conducted in accordance with the principles of the Declaration of Helsinki, as revised in 2013. Reporting follows the guidelines for Strengthening the Reporting of Observational Studies in Epidemiology (STROBE) (von Elm et al. 2007).

### 2.1 Study Population

**FIGURE 1:**
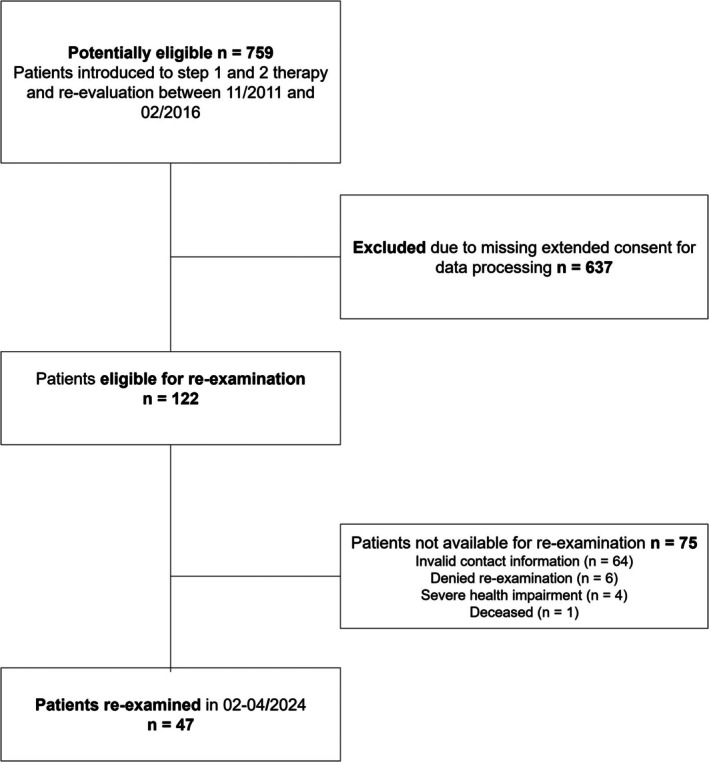
Flow chart of recruitment.

The initial study population comprised 759 patients who were recruited in a prospective trial between 2011 and 2016 (Figure 1). All of these patients were examined at baseline (T0) and received the first two steps of periodontal therapy as well as periodontal re‐evaluation in the undergraduate programme of the Department of Conservative Dentistry and Periodontology, University Hospital, LMU Munich. Re‐evaluation was performed 6.33 ± 3.79 months after step 2 of therapy (T1). Out of 122 patients of the initial cohort having agreed upon extended data processing after completion of the prospective trial, 47 patients were successfully re‐examined 126 ± 30 months after re‐evaluation between February and April 2024 (T2). All patients met the following inclusion criteria: (i) age ≥ 18 years; (ii) diagnosis of periodontitis according to the 2017 classification (Papapanou et al. 2018) at baseline; (iii) dental and periodontal charts providing at least probing pocket depth (PPD), plaque and bleeding on probing (BOP) at six sites/tooth, tooth mobility as well as furcation involvement (FI) at baseline and re‐evaluation; and (iv) consent to re‐examination and assessment of the OHRQoL using the OHIP‐14 questionnaire.

### 2.2 Clinical and Patient‐Related Outcome Variables at Baseline (T0) and Re‐Evaluation (T1)

Periodontal and dental examinations were conducted in the same manner at T0 and T1 by two experienced periodontists (C.E. and R.H.) who were calibrated in advance (inter‐rater reliability of periodontal probing, *κ* = 0.82), using the models introduced by Heym et al. (2018, 2016) (Bumm, Schwendicke, Heck, et al. 2024). In detail, PPD were determined to the nearest millimetre at six sites per tooth using a PCP‐12 periodontal probe. According to van der Weijden et al., BOP was assessed approximately 30 s after probing (Van der Weijden et al. 1994). The degree of mobility was assigned as described by Miller (1950). FI was measured with a 2N Nabers probe and graded according to Hamp et al. (1975). Plaque was recorded at six sites according to O'Leary et al. (1972). Periodontal pockets were defined as PPD > 3 mm at T0 and as PPD = 4 mm with BOP or ≥ 5 mm at T1/T2 according to the latest classification (Chapple et al. 2018).

The following endpoints of therapy were established at the patient level at T1:

1. EFP: No periodontal pockets > 4 mm with BOP or no deep periodontal pockets (≥ 6 mm) (Sanz, Herrera, et al. 2020),
2. T2T: ≤ 4 sites with PPD ≥ 5 mm (Feres et al. 2020).

### 2.3 Active Periodontal Therapy

APT was performed by undergraduate students under the supervision of C.E. and R.H. and has been described in detail elsewhere (Bumm, Schwendicke, Heck, et al. 2024; Werner, Heck, et al. 2024; Bumm, Schwendicke, Pitchika, et al. 2024). Briefly, following step 1, which included detailed information on the aetiology, pathogenesis, risk factors and treatment of periodontitis as well as oral hygiene instructions and professional mechanical plaque removal, non‐surgical periodontal therapy (NSPT) was performed under local anaesthesia at all sites with PPD > 3mm using sonic devices and curettes. Selective re‐instrumentation was performed by C.E. or R.H. in case of improper debridement on the same appointment in order to maintain a consistent level of therapy quality. Residual pockets persisting after steps 1 and 2 received non‐surgical re‐instrumentation (NSRI) in the same manner as NSPT (Bumm, Schwendicke, Pitchika, et al. 2024). No surgical treatment of residual pockets was performed.

### 2.4 Clinical and Patient‐Reported Outcome Variables at Re‐Examination (T2)

Patients were recalled and examined 126 ± 30 months after T1 by a postgraduate student (S.G.) as part of her doctoral theses. Clinical measurements were performed as described for T0 and T1 and conducted by S.G. as well as two additional examiners (F.N. and I.F.), thus ensuring consistency of the collected data. OHRQoL was measured using the German short‐version of the OHIP‐14. Additionally, the prosthetic status of Stage IV patients was recorded, distinguishing between upper and lower jaw, fixed or removable and full or partial arch prostheses. Furthermore, the patient‐related variables age, gender, current smoking status and diabetes mellitus, as well as adherence to SPT and self‐reported tooth migration, were recorded.

SPT intervals were assessed using periodontal risk assessment (PRA) (Lang and Tonetti 2003). A binary variable was created, indicating sufficient or insufficient compliance with individually calculated intervals.

### 2.5 Statistical Analysis and Sources of Bias

Comprehensive details on statistical analyses, sample size calculation and potential sources of bias are provided in Appendix A of the online version of the *Journal of Clinical Periodontology*.

## 3 Results

### 3.1 Patient Characteristics

This study included 47 patients with an average age of 71.7 ± 10.5 years at re‐examination, of whom 53.2% were male (Table 1). The prevalence of smoking within the sample was low; 8.5% were identified as current smokers, and 12.8% reported a diagnosis of diabetes. Periodontal disease severity was classified as Stage III in 40.4% of patients and Stage IV in 59.6%, with 68.1% of the participants showing generalised forms. Regarding periodontal grading, 4.2% of patients were classified as Grade A, 66.0% as Grade B and 29.8% as Grade C. Considering the prosthetic status of Stage IV patients, 46.4% presented with fixed, 39.3% with removable and 14.3.% without any type of prostheses (Table 1). Also, 48.9% of patients were compliant with SPT recommendations and 51.1% showed irregular attendance. During NSPT, five patients (11.6%) received adjunctive systemic antibiotics: amoxicillin (500 mg) and metronidazole (400 mg), or metronidazole (400 mg) alone in cases of intolerance to penicillin derivatives, each prescribed three times a day for 7 days. Twelve participants (25.5%) reported tooth migration during the observation period. None of these patient characteristics differed significantly when comparing the achievement of the different clinical endpoints following the first two steps of therapy.

**TABLE 1:** Patient characteristics.

Variable	All (*n* = 47)	EFP (*n* = 1)	T2T (*n* = 16)	No EP (*n* = 30)	*p*
Age, years	71.7 ± 10.5	67.0	75.8 ± 7.5	69.6 ± 11.4	0.143
Male, *n* (%)	25 (53.2)	0 (0.0)	6 (37.5)	19 (63.3)	0.092
Smokers, *n* (%)	4 (8.5)	0 (0.0)	1 (6.3)	3 (10.0)	1.000
Cigarettes per day	14.3 ± 7.3	—	12	15 ± 8.7	0.792
Diabetes, *n* (%)	6 (12.8)	0 (0.0)	0 (0.0)	6 (20.0)	0.196
HbA1c	6.72 ± 1.1	—	—	6.72 ± 1.1	—
Stage					
III, *n* (%)	19 (40.4)	1 (100.0)	5 (31.3)	13 (43.3)	0.340
IV, *n* (%)	28 (59.6)	0 (0.0)	11 (68.8)	17 (56.7)	
Extent					
Generalised	32 (68.1)	1 (100.0)	10 (62.5)	21 (70.0)	0.825
Localised	15 (31.9)	0 (0.0)	6 (37.5)	9 (30.0)	
Grade					
A, *n* (%)	2 (4.3)	0 (0.0)	1 (6.3)	1 (3.3)	0.336
B, *n* (%)	31 (66.0)	0 (0.0)	12 (75.0)	19 (63.3)	
C, *n* (%)	14 (29.8)	1 (100.0)	3 (18.8)	10 (33.3)	
Prosthetic status (in Stage IV)					
Upper jaw					
None	4 (14.3)	0 (0.0)	2 (18.2)	2 (11.8)	0.219
Full arch removable	8 (28.6)	0 (0.0)	4 (36.4)	4 (23.5)	
Full arch fixed	1 (3.6)	0 (0.0)	0 (0.0)	1 (5.9)	
Partially removable	2 (7.1)	0 (0.0)	2 (18.2)	0 (0.0)	
Partially fixed	13 (46.4)	0 (0.0)	3 (27.3)	10 (58.8)	
Lower jaw					
None	5 (17.9)	0 (0.0)	2 (18.2)	3 (17.6)	0.730
Full arch removable	5 (17.9)	0 (0.0)	2 (18.2)	3 (17.6)	
Full arch fixed	0 (0.0)	0 (0.0)	0 (0.0)	0 (0.0)	
Partially removable	5 (17.9)	0 (0.0)	3 (27.3)	2 (11.8)	
Partially fixed	13 (46.4)	0 (0.0)	4 (36.4)	9 (52.9)	
Number of teeth at T0	26 [21;27]	26	24 [21;27]	26 [22;28]	0.715
Number of teeth at T2	23 [19;26]	26	22 [17;26]	24 [20;27]	0.321
Tooth loss between T0 and T2	1 [0;3]	0 [0;0]	2 [0;4]	1 [0;2]	0.241
Median probing depth T0, mm	2.0 [2.0;3.0]	2.0 [2.0;3.0]	2.0 [2.0;3.0]	2.3 [2.0;3.0]	0.488
Median probing depth T1, mm	2.0 [2.0;2.0]	2.0 [2.0;2.0]	2.0 [2.0;2.0]	2.0 [2.0;2.0]	0.765
Median probing depth T2, mm	3.0 [2.0;3.0]	2.0 [2.0;2.0]	2.3 [2.0;3.0]	3.0 [2.0;3.0]	0.245
Proportion of sites with periodontal pockets at T0, %	14.0 [7.6;25.0]	5.1	9.9 [6.3;23.5]	15.6 [8.5;31.9]	0.212
Proportion of sites with periodontal pockets at T1, %	7.6 [4.8;13.3]	7.7	5.6 [1.9;9.7]	8.1 [5.6;15.4]	0.129
Proportion of sites with periodontal pockets at T2, %	10.6 [6.7;19.0]	0.6	6.4 [2.0;9.3]	16.6 [9.6;24.6]	< 0.001
Proportion of sites with deep periodontal pockets at T0, %	3.2 [0.0;9.1]	0.6	2.2 [0.0;10.0]	4.3 [0.4;9.5]	0.610
Proportion of sites with deep periodontal pockets at T1, %	1.4 [0.0;4.3]	1.3	0.0 [0.0;2.1]	1.7 [0.2;4.9]	0.195
Proportion of sites with deep periodontal pockets at T2, %	1.5 [0.0;4.5]	0.0	0.0 [0.0;0.8]	2.9 [1.4;7.6]	< 0.001
Not reached EFP at T1 and T2, *n* (%)	47 (100.0)	1 (100.0)	16 (100.0)	30 (100.0)	—
Not reached T2T at T1 and T2, *n* (%)	38 (80.9)	0 (0.0)	8 (50.0)	30 (100.0)	< 0.001
Systemic antibiotics as adjunct to step 2	5 (11.6)	0 (0.0)	3 (21.4)	2 (7.1)	0.370
AMX + MET	3 (7.0)	0 (0.0)	2 (13.3)	1 (3.7)	0.778
MET	2 (4.7)	0 (0.0)	1 (6.7)	1 (3.7)	
Patients with tooth mobility ≥ 2	12 (25.5)	0 (0.0)	3 (18.8)	9 (30.0)	0.626
Tooth migration	12 (25.5)	0 (0.0)	3 (18.8)	9 (30.0)	0.626
SPT regular	23 (48.9)	1 (100.0)	5 (31.3)	17 (56.7)	0.152

*Note*: Continuous data are presented as mean ± standard deviation or median [q1;q3]; Nominal data are presented as *n* (%). Comparisons are performed using analysis of variance, Kruskal–Wallis test or Fisher's exact test. Periodontal pockets were defined as probing pocket depth ≥ 4 mm at T0 and PPD = 4 mm with BOP or PPD ≥ 5 mm at T1. Deep periodontal pockets were defined as probing pocket depth ≥ 6 mm; SPT regular (≥ 2 visits/year). Statistical comparison were performed for exploratory purpose, adjustment of *p*‐values for false discovery rate was not considered.

Abbreviations: AMX, amoxicillin 500 mg t.i.d., 7 days; EFP, endpoint defined by Sanz et al.; EP, endpoint; MET, metronidazole 400 mg t.i.d., 7 days; OHIP, oral health impact profile; SPT, supportive periodontal therapy; T0, before steps 1 and 2 therapy; T1, 6.33 ± 3.79 months after step 2 therapy; T2, 126 ± 30 months after step 2 therapy; T2T, endpoint defined by Feres et al.

### 3.2 Clinical Endpoints and Periodontal Stability

Following steps 1 and 2, one patient achieved the EFP endpoint, 16 achieved T2T and 30 patients failed to achieve the endpoint of therapy, irrespective of the definition (Table 1). The median number of teeth decreased from 26 (21–27) at T0 to 23 (19–26) at T2, with a median TL between T0 and T2 of 1 (0–3). The proportion of sites with periodontal pockets (PPD > 3 mm) at T0 was 14.0%, which decreased to 7.6% by T1 (PPD = 4 mm with BOP or ≥ 5 mm) and increased slightly to 10.6% at T2. Similarly, deep periodontal pockets (≥ 6 mm) initially decreased from 3.2% at T0 to 1.4% at T1, followed by a slight increase to 1.5% at T2. Patients failing the endpoints of therapy showed significantly higher proportions of sites with periodontal pockets and especially deep periodontal pockets at T2 compared to those having achieved either the EFP endpoint or the T2T at T1 (Table 1). Endpoint analysis at T2 showed that a majority of the cohort did not meet clinical endpoints associated with periodontal stability over the observed period (Table 1).

### 3.3 OHIP‐14 Scores and Quality of Life

Patient characteristics and the corresponding OHIP‐14 scores with subdomains are shown in Table 2. The overall OHIP‐14 score at T2 averaged 4.87 ± 6.38, indicating a generally low impact on QoL across the cohort. The analysis of subdomains revealed functional limitation scores of 0.47 ± 0.78, with minimal issues reported in areas such as pronouncing words and taste alterations. Handicap scores, indicating general life satisfaction, averaged 0.45 ± 0.88, with most patients (83.0%) not finding life less satisfying due to oral health, although 12.8% reported occasional dissatisfaction.

**TABLE 2:** Patient characteristics and corresponding OHIP scores.

Variable	No. of patients (%)	Total OHIP‐G14	OHIP scores of sub‐domains						
			Functional limitation	Handicap	Psychological disability	Psychological discomfort	Physical disability	Physical pain	Social disability
Total	47	4.87 ± 6.38	0.47 ± 0.78	0.45 ± 0.88	0.83 ± 1.31	1.21 ± 1.59	0.40 ± 0.88	1.09 ± 1.64	0.43 ± 0.83
Sex									
Male	25 (53.2)	4.28 ± 5.44	0.56 ± 0.87	0.28 ± 0.61	0.76 ± 1.20	1.00 ± 1.41	0.32 ± 0.69	0.88 ± 1.30	0.48 ± 0.82
Female	22 (46.8)	5.55 ± 7.37	0.36 ± 0.66	0.64 ± 1.09	0.91 ± 1.44	1.45 ± 1.77	0.50 ± 1.06	1.32 ± 1.96	0.36 ± 0.85
Smoking status									
Non‐smoking	43 (91.5)	4.58 ± 6.49	0.33 ± 0.61	0.47 ± 0.91	0.84 ± 1.36	1.19 ± 1.58	0.35 ± 0.87	1.05 ± 1.63	0.37 ± 0.79
Smoking	4 (8.5)	8.00 ± 4.55	2.00 ± 0.82	0.25 ± 0.50	0.75 ± 0.50	1.50 ± 1.91	1.00 ± 0.82	1.50 ± 1.91	1.00 ± 1.15
Stage									
III	19 (40.4)	3.53 ± 4.86	0.32 ± 0.48	0.32 ± 0.68	0.68 ± 1.20	0.89 ± 1.20	0.26 ± 0.73	0.84 ± 1.21	0.21 ± 0.53
IV	28 (59.6)	5.79 ± 7.17	0.57 ± 0.92	0.54 ± 1.00	0.93 ± 1.39	1.43 ± 1.79	0.50 ± 0.96	1.25 ± 1.88	0.57 ± 0.96
Grade									
A	2 (4.3)	13.50 ± 7.78	1.00 ± 1.41	2.00 ± 0.00	2.00 ± 0.00	3.00 ± 1.41	1.00 ± 1.41	3.00 ± 1.41	1.50 ± 2.12
B	31 (66.0)	4.29 ± 6.01	0.26 ± 0.51	0.45 ± 0.93	0.77 ± 1.33	1.26 ± 1.67	0.29 ± 0.78	0.90 ± 1.56	0.35 ± 0.71
C	14 (29.8)	4.93 ± 6.62	0.86 ± 1.03	0.21 ± 0.58	0.79 ± 1.31	0.86 ± 1.29	0.57 ± 1.02	1.21 ± 1.76	0.43 ± 0.85
SPT attendance									
SPT regular	23 (48.9)	4.22 ± 5.13	0.43 ± 0.79	0.43 ± 0.73	0.61 ± 0.84	1.00 ± 1.38	0.43 ± 0.95	0.93 ± 1.31	0.39 ± 0.72
SPT irregular	24 (51.1)	5.50 ± 7.44	0.50 ± 0.78	0.45 ± 1.02	1.04 ± 1.63	1.04 ± 1.77	0.38 ± 0.83	1.25 ± 1.92	0.46 ± 0.93
Prosthetic status (in Stage IV)									
Fixed	13 (46.4)	6.08 ± 7.97	0.85 ± 1.07	0.69 ± 1.25	0.85 ± 1.40	1.23 ± 1.92	0.54 ± 0.97	1.39 ± 2.26	0.54 ± 1.05
Removeable	11 (39.3)	3.27 ± 4.03	0.09 ± 0.30	0.27 ± 0.65	0.45 ± 0.69	1.09 ± 1.38	0.27 ± 0.90	0.82 ± 1.17	0.27 ± 0.47
None	4 (14.3)	11.75 ± 9.14	1.00 ± 1.15	0.75 ± 0.96	2.50 ± 1.91	3.00 ± 2.00	1.00 ± 1.15	2.00 ± 2.31	1.50 ± 1.29
Endpoint									
EFP	1 (2.1)	0.00 ± 0.00	0.00 ± 0.00	0.00 ± 0.00	0.00 ± 0.00	0.00 ± 0.00	0.00 ± 0.00	0.00 ± 0.00	0.00 ± 0.00
T2T	16 (34.0)	6.44 ± 7.62	0.44 ± 0.73	0.69 ± 1.14	1.31 ± 1.85	1.88 ± 1.78	0.31 ± 0.87	1.31 ± 2.02	0.50 ± 0.89
None	30 (63.8)	4.20 ± 5.63	0.50 ± 0.82	0.33 ± 0.71	0.60 ± 0.86	0.90 ± 1.40	0.47 ± 0.90	1.00 ± 1.44	0.40 ± 0.81
Stability (endpoints at T1 and T2)									
Yes	9 (19.1)	6.67 ± 8.97	0.22 ± 0.44	0.67 ± 1.41	1.56 ± 2.12	2.00 ± 2.06	0.33 ± 1.00	1.44 ± 2.46	0.44 ± 1.01
No	38 (80.9)	4.45 ± 5.67	0.52 ± 0.83	0.39 ± 0.72	0.65 ± 0.99	1.03 ± 1.42	0.42 ± 0.86	1.00 ± 1.41	0.42 ± 0.79

*Note*: Continuous data are presented as mean ± standard deviation. Nominal data are presented as *n* (%). SPT attendance according to individual periodontal risk assessment (PRA).

Abbreviations: EFP, endpoint defined by Sanz et al.; OHIP, oral health impact profile; SPT, supportive periodontal therapy; T1, 6.33 ± 3.79 months after step 2 therapy; T2, 126 ± 30 months after step 2 therapy T2T, endpoint defined by Feres et al.

The psychological dimensions of OHRQoL revealed moderate impacts. The psychological disability subdomain, which assesses patients' difficulty in relaxing and feelings of embarrassment, had an average score of 0.83 ± 1.31. Similarly, psychological discomfort, capturing tension and self‐consciousness, averaged 1.21 ± 1.59. These scores suggest that while many patients did not experience substantial psychological impacts, a subset faced notable emotional challenges related to their oral health status.

Physical disability, which includes limitations on dietary choices and interruptions in meals, scored an average of 0.40 ± 0.88. Physical pain, assessing discomfort while eating and painful aching, had a higher mean score of 1.09 ± 1.64, with approximately 23.4% of patients reporting occasional pain. Social disability, reflecting difficulties in social interactions or daily tasks, scored 0.43 ± 0.83, suggesting that social impacts were generally minor but affected some patients (Table 2).

#### 3.3.1 Subgroup Analysis by Demographics and Clinical Characteristics

A subgroup analysis by demographic and clinical characteristics (Table 2) revealed lower OHIP‐14 scores in male (4.28 ± 5.44) than in female participants (5.55 ± 7.37), with specific increases in psychological discomfort and physical pain. Smoking status also showed a notable association with OHRQoL, with smokers reporting higher scores (8.00 ± 4.55) compared to non‐smokers (4.58 ± 6.49), especially in the subdomains physical discomfort and social disability.

Comparing the severity of disease, Stage IV patients reported higher overall OHIP‐14 scores (5.79 ± 7.17) than those with Stage III (3.53 ± 4.86). Stage IV patients experienced more psychological discomfort and physical pain, consistent with advanced disease stages and related complications. Patients assigned Grade A showed mean OHIP‐14 scores of 13.50 ± 7.78, while Grade B and Grade C patients reported lower scores, with averages of 4.29 ± 6.01 and 4.93 ± 6.62, respectively (Table 2).

Regarding the prosthetic status of Stage IV patients, the analysis revealed that those not having received a prosthetic rehabilitation reported higher OHIP‐14 scores (11.75 ± 9.14) than those with fixed (6.08 ± 7.97) or removable (3.27 ± 4.03) prostheses.

Adherence to SPT also influenced QoL outcomes. Patients who were compliant with SPT visits had a mean OHIP‐14 score of 4.22 ± 5.13, compared to 5.50 ± 7.44 in non‐compliant patients. Non‐compliant patients reported higher scores in psychological discomfort, physical pain and social disability.

### 3.4 Regression Analysis

Multiple regression analyses (Table 3), adjusted for the potentially confounding variables sex, smoking, diabetes and SPT compliance, were performed using the OHIP‐14 score as the dependent variable. The models identified that neither the achievement of clinical endpoints following steps 1 and 2 therapy nor periodontal stability over the observation period, indicated by the achievement of clinical endpoints at both T1 and T2, was significantly associated with OHIP‐14 scores. Likewise, disease severity and adherence to SPT showed no significant association. Notably, self‐reported tooth migration was identified as a significant factor influencing OHIP‐14 scores at T2 in all regression models (*p* = 0.010). A subgroup analysis including Stage IV patients only, and thus accounting for prosthetic status, confirmed the findings of the prior regression model identifying tooth migration as the only significantly associated factor (Table S2).

**TABLE 3:** Linear regression models—dependent variable OHIP‐14 at T2.

Variables	Endpoint analysis at T1		Endpoint analysis at T2		Endpoint analysis at T1 and T2	
	ß‐Coefficient (95% CI)	*p*	ß‐Coefficient (95% CI)	*p*	*ß*‐Coefficient (95% CI)	*p*
	Adjusted for sex, smoking, diabetes and SPT compliance		Adjusted for sex, smoking, diabetes and SPT compliance		Adjusted for sex, smoking, diabetes and SPT compliance	
EFP or T2T vs. None	−1.631 (−6.319 to 3.057)	0.485	−2.320 (−6.526 to 1.886)	0.271	−3.173 (−8.462 to 2.117)	0.303
Stage III vs. IV	1.709 (−2.567 to 5.985)	0.423	1.812 (−2.132 to 5.755)	0.359	1.918 (−2.009 to 5.844)	0.300
Tooth migration	7.090 (1.899–12.280)	**0.009**	5.941 (1.515–10.368)	**0.010**	5.923 (1.528–10.317)	**0.010**

*Note*: Data are presented as β‐coefficient with corresponding 95% confidence interval (CI). Bold indicates statistically significant values (*p* < 0.05).

Abbreviations: EFP, endpoint defined by Sanz et al.; OHIP, oral health impact profile; SPT, supportive periodontal therapy; T1, 6.33 ± 3.79 months after step 2 therapy; T2, 126 ± 30 months after step 2 therapy; T2T, endpoint defined by Feres et al.

## 4 Discussion

### 4.1 Key Findings and Objective

The results of this longitudinal study suggest that achieving therapy endpoints, often regarded as indicators of treatment success and long‐term stability, was not significantly associated with patient‐perceived OHRQoL. Patients reporting on tooth migration during the observation period, on the other hand, revealed significantly worse OHRQoL. These findings align with existing literature, suggesting that traditional clinical metrics may not fully capture the nuanced ways periodontal disease affects patients' daily lives and satisfaction with their oral health.

### 4.2 Limitations

This study has several limitations that should be acknowledged. Although we conducted a longitudinal analysis of clinical parameters over three time points, OHRQoL was only assessed at T2, limiting the ability to track changes in QoL over time. As such, it remains unclear how OHRQoL evolved during the treatment process and whether certain clinical improvements correlated with temporary or lasting changes in perceived QoL. Future studies with repeated OHRQoL assessments could provide a clearer picture of the progression of patient perceptions alongside clinical outcomes.

Considering the monocentric, non‐controlled nature of the study as well as the sample size of 47 patients and the high mean age of the participants, generalisability of the present findings is not possible. In the present study, periodontal treatment was performed by undergraduate students. To address this potential limitation, supervision of treatment and selective intervention in case of improper debridement during NSPT and NSRI were performed by two specialised periodontists (C.E., R.H.). In addition, our results on tooth migration and its association with OHRQoL must be interpreted with caution, as the self‐reported nature of this outcome variable might introduce bias related to the patients' individual perceptions, potentially leading to over‐ or under‐reporting. Future investigations should focus on establishing objective measures for tooth migration. Further, only one patient met the EFP endpoint following APT, reducing the statistical power of our data. To mitigate this limitation, we grouped the results of EFP and T2T endpoints for the regression models, depicted in Table 3 and S2. It must be further noted that statistical analysis of the variables in Table 1 was performed for exploratory purpose, hence adjustment of *p*‐values for multiple testing was not considered.

Although this sample provides valuable insights, larger cohorts would allow for more robust statistical analyses. Additionally, studies incorporating diverse patient populations could reveal how demographic and lifestyle factors influence long‐term OHRQoL after periodontal therapy.

### 4.3 Discussion of Methods and Results

The aim of this study was to investigate different endpoints of periodontal therapy on OHRQoL following long‐term SPT in elderly patients with severe periodontitis. There are several methods to measure patients' OHRQoL, with one of the most common ways being the use of generic questionnaires. While a more specific approach, as reflected by condition‐specific tools, seems reasonable to draw conclusions on the patient‐perceived OHRQoL, Rener‐Sitar et al. suggested that generic tools—OHIP in particular—may not only be sufficient in reflecting patients' OHRQoL but even be superior to condition‐specific tools, as they capture all relevant dimensions of oral health and make easy comparability of research data (Rener‐Sitar et al. 2021). Accordingly, in this study, a German short version of the OHIP (OHIP‐14) (John et al. 2006) was used, as suggested by Graetz et al. (2020). Although more comprehensive versions of the OHIP are available and used occasionally (El Sayed et al. 2019), John et al. demonstrated high correlation between the different versions and concluded that short versions are sufficient to reflect the patients' OHRQoL (John et al. 2022).

Regarding the investigated endpoints, only one patient achieved the endpoint suggested by the EFP (Sanz, Herrera, et al. 2020), while 34% met the T2T proposed by Feres et al. (2020), whereas 64% failed to achieve any endpoint following steps 1 and 2. It must be noted that the study cohort comprised mainly Stage III and Stage IV patients, in whom complete resolution of disease after initial therapy is known to be rare (Feres et al. 2020; Werner, Heck, et al. 2024; Yang et al. 2023; Citterio et al. 2022). Therefore, the high number of patients not achieving the EFP endpoint is not very surprising. The less stringent and more patient‐centred T2T endpoint has recently gained increasing recognition by periodontal researchers, as it may allow for a more objective comparison of studies as well as extrapolation of research data to clinical practice (Feres et al. 2020). Considering the short investigation period of 2 years published by Feres et al., it should, however, be noted that long‐term validation of this endpoint is needed. The results on this endpoint are in line with a recently published study of our group reporting 41% of patients achieving the T2T (Werner, Frasheri, et al. 2024). Although slightly higher, our findings can also be considered in accordance with a recently published study by Benz et al. (Benz et al. 2023), reporting a T2T rate of 27%. Notably, the subgroup having received antibiotics adjunctive to step 2 showed no significant differences regarding the achievement of endpoints at T1 or T2. While this partially contrasts with recent evidence suggesting significant changes in microbiota (Hagenfeld et al. 2023) and clinical outcomes (Teughels et al. 2020) following adjunctive antibiotic therapy, it should be emphasised that these findings are based on studies with follow‐up restricted to approximately 2 years, underlining the need for longer observation periods to address this sensitive topic.

Interestingly, regardless of adjunctive therapies, TL rates were low and did not differ significantly between participants who met or failed the endpoints. In terms of tooth retention, it might therefore be assumed that the reliability of the investigated endpoints to reflect periodontal stability for an observation period of 10 years is limited. Especially in this context, it should be questioned whether clinical endpoints suggesting certain thresholds of periodontal pocketing, clearly valuable to assess the quality of APT, are also capable of reflecting long‐term periodontal stability, or whether longitudinal data of attachment levels might be more suitable to address this aim.

Regarding patients' perceptions of oral health following long‐term SPT, our results were in line with published data, suggesting an overall low impact of periodontal disease and therapy on OHRQoL. In even longer observation periods, El Sayed et al. (2019) demonstrated that patients showed satisfactory oral health conditions and low OHRQoL impacts two decades post therapy. Likewise, Graetz et al. (2020) reported generally low OHRQoL impacts even 27 years after periodontal therapy. Notably, both of these studies included surgical interventions during APT, while the present cohort was treated strictly non‐surgically. Interestingly, given the similarly low impact of periodontal therapy on OHRQoL, it might be assumed that the modality of therapy (surgical/non‐surgical) is of less importance in this context.

The findings of El Sayed et al. and Graetz et al. indicate that, despite clinical improvements, residual concerns such as tooth mobility and aesthetic issues can affect patients' QoL over the long term. These findings are further supported by the current results, indicating that particularly tooth migration, as a persistent functional and aesthetic issue in advanced periodontitis patients, negatively impacts the patients' OHRQoL. The significant impact of tooth migration on OHRQoL observed in this study highlights the necessity of addressing functional and aesthetic concerns in periodontal care, particularly for patients with advanced periodontitis. According to the OHIP‐14 scores from our dataset, patients with tooth migration reported increased challenges in daily activities such as chewing, alongside psychological discomfort related to their appearance. These observations align with the findings of El Sayed et al. (2019), who reported that physical pain and functional limitations remained the most common impairments in long‐term periodontal patients, despite overall clinical stability.

The aesthetic concerns related to tooth migration are supported by Graziani and Tsakos (2020), who discussed the broader psychosocial impact of periodontal disease, including patients' self‐confidence and social interactions. As our data suggest, even in cases where clinical measures like PPD and BOP are within target ranges, patients may still perceive their oral health as compromised due to visible signs of disease, such as tooth migration. Addressing these aesthetic concerns through early interventions or adjunctive treatments, such as orthodontic therapy or aesthetic restorations, may be crucial for improving patient satisfaction with periodontal outcomes.

The lack of a strong association between the investigated clinical endpoints and OHRQoL in this cohort aligns with a growing body of evidence advocating for a more patient‐centred approach in periodontal care (Needleman et al. 2023). In this context, Graziani et al. have emphasised that QoL measures such as OHIP‐14 capture a wider array of patient experiences, encompassing functional limitations, psychological discomfort and social disability (Graziani and Tsakos 2020). It is important to mention that neither the EFP endpoint nor the T2T was intended to assess patient‐related outcomes; therefore, by utilising patient‐reported outcome measures in addition to clinical assessments, clinicians may gain a more comprehensive understanding of treatment success from the patient's perspective. This approach is particularly relevant in our cohort, where tooth migration emerged as a significant factor in perceived oral health quality, despite meeting clinical treatment targets.

## 5 Conclusion

In conclusion, this study suggests that while clinical endpoints of periodontal therapy, such as achieving target PPD or BOP levels, are critical to monitor and maintain attachment levels, they may not fully reflect patients' perceptions of their individual oral health. Tooth migration, as both a functional and aesthetic concern that is not reflected by clinical endpoints of therapy, significantly affected OHRQoL. These findings underscore the need for a more holistic approach in periodontal care that integrates both clinical and patient‐reported outcomes to fully capture the impact of treatment on long‐term QoL. By acknowledging and addressing patient‐specific concerns, periodontal therapy can become more tailored, ultimately improving patient satisfaction and QoL outcomes.

## Author Contributions

C.V.B., F.S., M.F. and N.W. contributed to the study design and conception, to data analysis and interpretation and to the writing and revision of this manuscript. S.G., F.N., I.F., C.W., C.E. and R.H. contributed to data collection and to the writing and revision of this manuscript. V.P. contributed to data analysis and interpretation, and to the writing and revision of this manuscript. All authors reviewed and approved the final manuscript and agreed to be accountable for all aspects of work ensuring integrity and accuracy.

## Ethics Statement

This longitudinal follow‐up study was approved by the Ethics Committee of the Medical Faculty of the Ludwig‐Maximilians‐University, Munich (025‐11).

## Conflicts of Interest

The authors declare no conflicts of interest.

## Supporting information


**Table S1.** Baseline data of dropped out patients.
**Table S2**. Subgroup analysis for Stage IV patients: Linear regression models—dependent variable OHIP‐14 at T2.

## Data Availability

The data that support the findings of this study are available from the corresponding author upon reasonable request.

## Appendix A

### Statistical Analysis

Normal distribution of data was tested using the Kolmogorov–Smirnov test. Differences between different endpoints were compared using analysis of variance (ANOVA) for continuous variables, the Kruskal–Wallis test for ordinal and skewed variables and Fisher's exact test for categorical variables. Post hoc pairwise analysis for the Kruskal–Wallis test was done using the Dunn–Bonferroni test. Data were expressed as mean ± standard deviation (SD) or median with interquartile ratio [q1;q3], and categorical variables were presented as frequencies (with percentage). Linear regression was used to model the association between OHRQoL and endpoints adjusted for confounders (sex, smoking, diabetes and SPT compliance). To increase the robustness of the assessment, the two investigated endpoints (EFP/T2T) were merged for regression analyses. Results are shown as adjusted β‐coefficient per 1‐unit change of OHIP‐14 with corresponding 95% confidence intervals (CIs). The significance level was set at α = 0.05 for all tests. All analyses were performed using SPSS (Version 29.0, IBM, Armonk, USA).

### Sample Size

A formal a priori sample size calculation was not performed. However, power analyses were conducted based on the *R* ^2^ values derived from the regression models (Table 3) described in the Results section (0.244, 0.232 and 0.243). To streamline the analysis, an average *R* ^2^ value (0.240) was used. Other parameters for the power calculation included the number of predictors, a significance level of 0.05 and the study sample size. Using these parameters, the calculated power for the study reached 0.88.

### Sources of Bias

Several sources of bias regarding clinical measurements and therapeutic interventions as well as data interpretation and reporting were identified. To mitigate misclassification and performance bias that might have occurred during diagnosis or therapy, the supervising operators (C.E. and R.H.) were calibrated to ensure a consistent level of data collection. At re‐examination, clinical data were collected by three independent examiners (S.G., F.N., I.F.) reaching consensus in the event of deviating measurements and thus compensating for missing calibration with the initial examiners. To ensure a consistent level of therapeutic quality, selective re‐instrumentation was performed by C.E. or R.H. in case of improper debridement during NSPT and NSRI. In advance of the collection of the patient‐reported outcomes at T2, neither re‐examiners nor patients were provided information on the clinical outcomes observed at T0 or T1 (including whether endpoints were reached/missed) in order to reduce potential information bias. To address potential selection bias associated with the substantial drop‐out rate, a missing data analysis was performed by comparing key baseline characteristics between patients who were re‐examined and those lost to follow‐up (Table S1). No statistical differences were identified between the two groups, indicating a low risk of bias due to non‐random attrition.
