# Supplementary material for: Endpoints of Periodontal Therapy in Elderly Patients With Stage III/IV Periodontitis and Their Oral Health–Related Quality of Life Following 10 Years of Supportive Periodontal Therapy
Source: J Clin Periodontol. 2025 Jul 6;52(10):1398–409. doi: 10.1111/jcpe.14198 (PMC12420083; doi:10.1111/jcpe.14198)
Supplement: Supplementary file 1 — Table S1. Baseline data of dropped out patients. Table S2. Subgroup analysis for Stage IV patients: Linear regression models—dependent variable OHIP‐14 at T2. [file JCPE-52-1398-s001.docx]

**Endpoints of Periodontal Therapy in Elderly Patients with Stage III/IV Periodontitis and their Oral Health-related Quality of Life following 10 Years of Supportive Periodontal Therapy**

-Supplementary Tables-

| Table S1: Baseline data of drop out patients | | |  |
| --- | --- | --- | --- |
| Variable | N=75 | N=47 | p-value |
| Age, years | 61.55±10.4 | 60.19±10.5 | 0.514 |
| Male, n (%) | 38 (48.7) | 25 (53.2) | 0.842 |
| Smokers, n (%) | 13 (16.7) | 4 (8.5) | 0.127 |
| Diabetes, n (%) | 7 (7.7) | 6 (12.8) | 0.849 |
| Systemic antibiotics in adjunction to step 2 | 11 (15.9) | 5 (11.6) | 0.526 |
| AMX + MET | 4 (6.0) | 3 (7.0) | 0.597 |
| MET | 7 (10.4) | 2 (4.7) |  |
| Proportion of sites with periodontal pockets at T0, % | 17.9 [9.6;33.8] | 14.0 [7.6;25.0] | 0.135 |
| Proportion of sites with deep periodontal pockets at T0, % | 2.7 [0.6;10.8] | 3.2 [0.0;9.1] | 0.708 |

AMX, amoxicillin 500 mg t.i.d., 7 days; MET, metronidazole 400 mg t.i.d., 7 days

| Table S2: Subgroup analysis for stage IV patients: Linear regression models – dependent variable OHIP-14 at T2 | | | | | | |
| --- | --- | --- | --- | --- | --- | --- |
| Variables | **Endpoint analysis at T1** | | **Endpoint analysis at T2** | | **Endpoint analysis at T1 and T2** | |
|  | ß-coefficient (95 % CI) Adjusted for sex, smoking, diabetes and SPC compliance | p-value | ß-coefficient (95 % CI) Adjusted for sex, smoking, diabetes and SPC compliance | p-value | ß-coefficient (95 % CI) Adjusted for sex, smoking, diabetes and SPC compliance | p-value |
| EFP or T2T vs None | -0.941 (-9.087;7.205) | 0.810 | -3.746 (-7.509; 2.518) | 0.828 | -4.777 (-9.984;2.682) | 0.196 |
| Removeable rehabilitation vs no rehabilitation | -5.404 (-16.298;5.490) | 0.309 | -5.014 (-14.036;4.008) | 0.226 | -5.132 (-14.080;3.816) | 0.245 |
| Fixed rehabilitation vs no rehabilitation | -4.267 (-15.184;6.649) | 0.419 | -4.116 (-13.301;5.068) | 0.259 | -5.166 (-14.283;3.952) | 0.250 |
| Tooth migration | 8.346 (-0.443;17.135) | 0.061 | 8.623 (1.521;15.726) | **0.020** | 8.146 (1.273;15.019) | **0.023** |
| Data are presented as β-coefficient with corresponding 95% confidence interval (CI) EFP, endpoint defined by Sanz et al.; T2T, endpoint defined by Feres et al; OHIP, oral health impact profile; T1, 6.33 ± 3.79 months after step 2 therapy; T2, 126 ± 30 months after steps 1 and 2 therapy  Bold indicates statistically significant values (p < 0.05). | | | | | | |
